# Evidence of transovarial transmission of Chikungunya and Dengue viruses in field-caught mosquitoes in Kenya

**DOI:** 10.1371/journal.pntd.0008362

**Published:** 2020-06-19

**Authors:** Claire J. Heath, Elysse N. Grossi-Soyster, Bryson A. Ndenga, Francis M. Mutuku, Malaya K. Sahoo, Harun N. Ngugi, Joel O. Mbakaya, Peter Siema, Uriel Kitron, Nayer Zahiri, Jimmy Hortion, Jesse J. Waggoner, Charles H. King, Benjamin A. Pinsky, A. Desiree LaBeaud

**Affiliations:** 1 Stanford University School of Medicine, Stanford, California, United States of America; 2 Kenya Medical Research Institute, Kisumu, Kenya; 3 Technical University of Mombasa, Mombasa, Kenya; 4 Chuka University, Chuka, Kenya; 5 Vector Borne Disease Unit, Msambweni, Kenya; 6 Emory University, Atlanta, Georgia, United States of America; 7 San Mateo County Mosquito and Vector Control District, Burlingame, California, United States of America; 8 Case Western Reserve University, Cleveland, Ohio, United States of America; University of Wisconsin Madison, UNITED STATES

## Abstract

Arboviruses are among the most important emerging pathogens due to their increasing public health impact. In Kenya, continued population growth and associated urbanization are conducive to vector spread in both urban and rural environments, yet mechanisms of viral amplification in vector populations is often overlooked when assessing risks for outbreaks. Thus, the characterization of local arbovirus circulation in mosquito populations is imperative to better inform risk assessments and vector control practices. *Aedes* species mosquitoes were captured at varying stages of their life cycle during different seasons between January 2014 and May 2016 at four distinct sites in Kenya, and tested for chikungunya (CHIKV), dengue (DENV) and Zika (ZIKV) viruses by RT-PCR. CHIKV was detected in 45 (5.9%) and DENV in 3 (0.4%) mosquito pools. No ZIKV was detected. Significant regional variation in prevalence was observed, with greater frequency of CHIKV on the coast. DENV was detected exclusively on the coast. Both viruses were detected in immature mosquitoes of both sexes, providing evidence of transovarial transmission of these arboviruses in local mosquitoes. This phenomenon may be driving underlying viral maintenance that may largely contribute to periodic re-emergence among humans in Kenya.

## Author summary

Transovarial transmission, or vertical transmission, is the spread of a pathogen from parent to offspring. It has been observed that some mosquito-borne viruses can be transmitted from female mosquitoes to their offspring during follicle development or during oviposition. The occurrence of transovarial transmission is evident in the presence of virally infected male mosquitoes, which typically do not take bloodmeals, and the presence of virus in immature mosquitoes of any sex. Transovarial transmission aids in the amplification of mosquito-borne viruses in the environment by increasing the number of infected mosquitoes in a given region, thus expanding the possibility of viral transmission to humans. The combination of transovarial transmission and the preservation of viable eggs during dry seasons may trigger sudden amplification of the virus after rainy periods, resulting in an outbreak. This study provides some of the first evidence of transovarial transmission of chikungunya and dengue viruses in *Aedes aegypti* mosquitoes in Africa during interepidemic periods, which has important implications for local virus persistence and epidemic patterns.

## Introduction

Arthropod–borne viruses (arboviruses) comprise some of the most important emerging pathogens due to their geographic spread and increasing impact on vulnerable human populations. Over 100 arboviruses are known to cause pathology in humans, creating a significant global health burden, yet the transmission, epidemiology, and incidence of arbovirus-related human disease remain poorly defined, particularly in sub-Saharan Africa. Kenya has had multiple arbovirus outbreaks in the past 2 decades including yellow fever [1, 2], chikungunya [3], Rift Valley fever [4–6] and dengue fever [7], which have resulted in significant effects to local economies and community health [8–13]. Thus, the characterization of arboviral circulation in the rapidly changing Kenyan environments that support vector proliferation is essential to better inform human risk assessment and vector control practices.

Transovarial transmission (TOT) is a mechanism by which infective female mosquitoes pass the virus to their offspring via their eggs. TOT is believed to be a mechanism by which arboviruses maintain a local presence during environmental conditions that are adverse for mosquito proliferation, *e*.*g*. during dry seasons and winter. Quiescence of vertically infected mosquito eggs in response to environmental conditions may contribute to viral persistence or re-emergence within a region [14]. A recent review of modeling studies suggests that, for DENV, TOT is likely not an important mechanism of viral propagation in Southeast Asia and the Americas, but rather asymptomatic infections in humans and the movement of people account for DENV persistence [15]. Others theorize that selective pressures arise from different vector-virus system combinations that influence the success of horizontal and vertical transmission in a population, as well as overall virulence [16, 17]. Although comprehensive evidence exists demonstrating that TOT of CHIKV and DENV occurs in many distinct endemic regions, complex mechanisms and a lack of field data lead to an incomplete understanding of the contributions of TOT to viral persistence during interepidemic times. In particular, the propagation and maintenance of DENV and CHIKV in Africa and the role of TOT in this viral maintenance is not well understood. This study describes the prevalence and spatiotemporal distribution of CHIKV, DENV, and ZIKV in field-caught mosquitoes in Kenya. We further investigate the rate of TOT of these viruses to determine its possible contribution to viral maintenance in this endemic setting.

## Methods

### Ethics statement

The study protocol was approved by the Stanford University Institutional Review Board (Protocol ID #31488) and the Kenya Medical Research Institute (KEMRI) National Scientific and Ethical Review Committee (SSC # 2611). Meetings were held at all four sites with local government administrators (village elders, chiefs, and assistant chiefs) and with the local residents in each sub-location to introduce the research study and staff to the public. Written informed consent was obtained from the adults who volunteered to participate in Human Landing Catches (HLC) before they were trained and began sampling for mosquitoes. Oral consent was obtained from household heads to sample mosquitoes within their houses and their compounds.

### Mosquito collection

**Fig 1:**
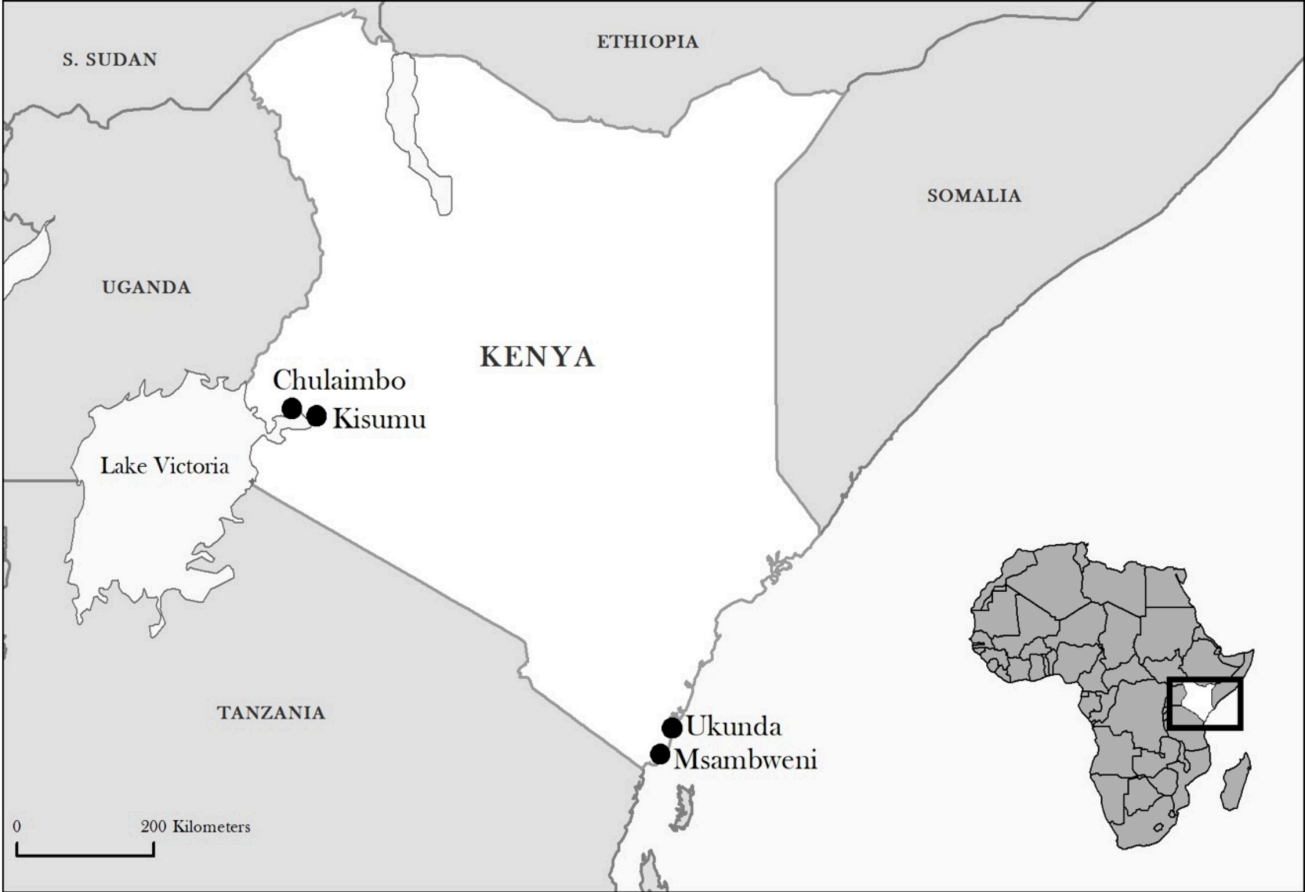
Map of study sites in Kenya. Created in QGIS 2.18.11 using MapBox.

Study sites: This study was conducted in four sites in Kenya: two western sites, Kisumu (urban) and Chulaimbo (rural) in Kisumu County, and two coastal sites, Ukunda (urban) and Msambweni (rural) in Kwale County (Fig 1). Kisumu is the third largest city in Kenya located at the shore of Lake Victoria. Chulaimbo village is located 19 kilometers from Kisumu. Ukunda is an emerging urban center located 30 kilometers south of Mombasa in Kwale County along the Indian Ocean coastline. Msambweni village is also in Kwale County, near the shore of the Indian Ocean. It is located 30 kilometers south of Ukunda nearer the Tanzanian border. The climate in both regions is tropical with a bimodal monthly rainfall pattern, with a long rainy period from March to June and a short rainy period from August to October. Sampling for *Aedes* mosquitoes in each of the four sites was done in a selected area of approximately 1.5 x 1.0 km.

The mosquito collection methods have been previously described in detail [18, 19] and are briefly summarized here:

Human Landing Catches (HLC): Two homesteads were selected in each of the sites for sampling of blood-seeking mosquitoes using HLC both indoors and outdoors. Paired teams worked together, with one exposing the legs, and the other collecting mosquitoes landing on their partner’s legs. Team members changed roles hourly. Captured mosquitoes were placed in collection vessels provided with 10% sugar solution on cotton wool. Vessels were transported on ice to insectaries at KEMRI at Kisian in Kisumu County (for the western sites) and the Vector Borne Disease Control Unit in Msambweni County Referral Hospital, Kwale County (for coastal sites).

Prokopack Automated Aspirators: Twenty houses were randomly selected in each of the sites and sampled indoors and outdoors monthly for resting mosquitoes using Prokopack aspirators [20]. Sampling was performed simultaneously, both indoors and outdoors, for 20 minutes by a pair of entomology team members. The collection vessel, which was fitted with a wire mesh on the underside, was used on the aspirator and was capped and removed at the end of each sampling period. Mosquito specimens were stored on ice and transported to each insectary as described above.

Biogents-Sentinel traps (BGS): Two houses were selected in each of the four sites for adult mosquito sampling using Biogents (BGS)-traps (Biogents AG Weissenburgstr 22 93055 Regensburg, Germany). Initially, the BGS traps were set indoors in the sitting room without lure. In May 2015, the houses were changed, the trap location was changed from indoors to open, secure verandas, and the traps were baited with CO_2_. The BGS trap was set to sample mosquitoes for five consecutive days every month. Trapped specimens were collected daily and stored and transported as above.

Sampling of immature mosquitoes: Pupal and Larval sampling: Forty houses in each of the four study sites were selected by random sampling: 20 houses for larval sampling and 20 for ovitrap sampling. Houses were assessed for immature mosquito infestation on a monthly basis over the study period. All natural and artificial water-holding containers in and around each household were inspected for mosquito larvae and pupae. All pupae and larvae (3^rd^ and 4^th^ instars) from positive containers were collected with pipettes and ladles [19, 21]. Water from large containers was first sieved and mosquito samples were placed in a white plastic tray with some water from which the immatures were pipetted. Mosquito samples were placed in 10mL Falcon tubes (Fisher Scientific, Waltham, MA) and/or Whirl-pak plastic bags (Nasco, Fort Atkinson, WI), labeled, and taken to the insectaries. There, immature mosquitoes were reared in 200 mL plastic cups under laboratory conditions at an average temperature of 28.15 ± 1.8ºC and relative humidity of 80.9 ± 6.3%, and larvae were fed on TetraMinbaby fish food until adult mosquitoes emerged.

Ovitrap Sampling: Ovitraps were placed in 20 households that were randomly selected as fixed sampling points in each of our four study sites. Each ovitrap consisted of a black plastic cup filled with 350 mL of tap, borehole, or rainwater. The inside of the cup was lined by a brown paper towel that was partially submerged. Eggs were laid on the damp paper towel just above the water line. Ovitraps were set once a week for the duration of the study. To obtain the eggs, the paper towel in each trap was removed, placed into a plastic storage bag and transported to the insectary facilities. Each paper towel was examined under the dissecting microscope for identification of *Aedes* species eggs. To confirm the species, the eggs were submerged in tap water for hatching and the larvae reared to adults under the conditions described above.

Mosquito identification: In the insectaries, once mature, all mosquitoes were killed by placing them at -20°C for 15 minutes. They were then sorted by genus (*Aedes* spp., *Anopheles* spp., or *Culex* spp.) and sex, using a standard identification key [22]. Further analyses described only detail data pertaining to *Aedes* spp. collected, with only *Ae*. *aegypti* specimens pooled and tested by methods described below.

Sample storage and transport for viral testing: A single leg was removed from each mosquito specimen and transferred to a 2mL Cryotube (Sarstedt, Rommelsdorf, Germany) with 0.5mL of RNA later (Sigma-Aldrich, St. Louis, MO) to preserve any viral nucleic acids. Samples were stored at -80°C until they were shipped to Stanford, CA, USA on dry ice for processing.

### Collection of rainfall data

To monitor rainfall at each of our sampling sites, one rain gauge (HOBO Onset data loggers, Onset Computer Corporation 470 Bourne, MA USA), was installed at each of the four sites to collect daily rainfall. The sites were: Chulaimbo Sub-District Hospital (Chulaimbo) (0°02'16.5"S, 34°38'20.1"E), Jaramogi Oginga Odinga Teaching and Referral Hospital (Kisumu) (0°05'17.5"S, 34°46'13.6"E), Msambweni County Referral Hospital (Msambweni) (4°28′12″S, 39°28′48″E) and Diani Health Centre (Ukunda) (4°16'44.5"S, 39°35'25.0"E). Data from rain gauges were downloaded monthly.

### Sample processing

Mosquito legs taken from mosquitoes that had been sorted by species, sex, trap type, and date of capture, were grouped into 714 pools of ~25 individual legs (range 1–36). Specimens of each type (sex etc.) were independently isolated, and all instruments were cleaned and decontaminated with 10% bleach solution between isolations to mitigate any potential for cross-contamination. Each pool of mosquito legs was then transferred into a 2.0 mL microcentrifuge tube with six 2 mm ceramic lysis beads (OMNI International GA USA) with 700 μL of MagMAX Lysis/Binding Solution Concentrate (Applied Biosystems, CA, USA). Samples were then homogenized in a Bead Ruptor 24 (OMNI International, GA, USA), according to the following program: Strength = 5.65, Time = 0:30, Cycles = 2, Delay = 0:05. Sample lysates were then centrifuged at 15,000 rpm for 6 minutes at room temperature to remove debris. Nucleic acid isolation was then performed using MAGMAX 96-Viral Isolation Kit (Life Technologies, CA, USA) according to the manufacturer’s instructions. The manufacturer’s decontamination protocol was performed after every run to prevent nucleic acid transfer between samples.

### Molecular testing

Pooled legs of *Aedes aegypti* mosquitoes were tested for the presence of ZIKV, CHIKV, and DENV RNA with a triplex real-time reverse transcription PCR (rRT-PCR), as previously described [23]. Briefly, ZIKV-CHIKV-DENV rRT-PCR reactions were performed using the SuperScript III Platinum One-Step qRT-PCR kit (Life Technologies) with a total reaction volume of 25 μL and 5 μL of eluate. Reactions were performed on a Rotor-Gene Q instrument (Qiagen). Thresholds were set according to the published validation, and all exponential curves that crossed the threshold prior to cycle 45 were considered positive [24]. Negative controls containing no RNA template were included on every run of the assay, and amplicon curves were not observed in any of these controls. To validate our assay results, we performed monoplex PCR assays, using the same primer sets included in the multiplex reaction, on a subset of 18 CHIKV positive samples, and all 3 of the DENV positive samples to confirm infection.

### Statistical analysis

Multi-way chi-square (χ^2^) analyses were performed to determine statistical differences among the levels of positivity for the recovered samples from different trap types. Maximum likelihood estimates of viral infection rates were calculated using the Poolscreen methodology developed by Katholi and Unnasch [25].

## Results

### Prevalence of infection

Between January 2014 and May 2016 mosquitoes were collected using multiple trapping methods at 4 study sites in Kenya; Chulaimbo (rural) and Kisumu (urban) in the west of the country, and Msambweni (rural) and Ukunda (urban) on the coast (Fig 1).

**Fig 2:**
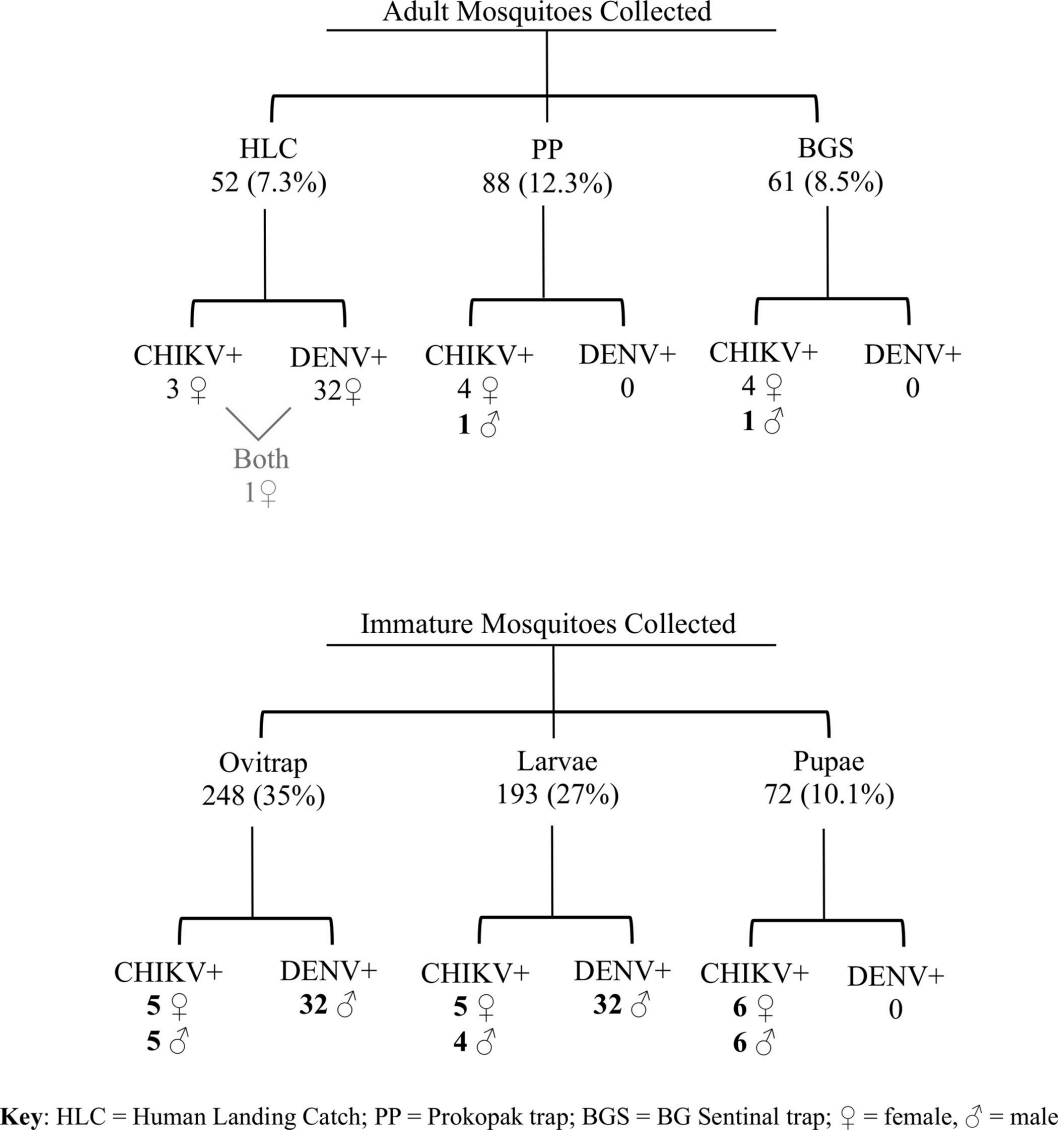
Flow chart describing pools of *Ae*. *aegypti* mosquito legs by age, sex, and virus detected. All mosquito leg pools described were collected from four distinct study sites: Kisumu and Chulaimbo (urban and rural sites, respectively, in western Kenya), and Ukunda and Msambweni (urban and rural sites, respectively, in coastal Kenya) between January 2014 and May 2016.

A total of 714 pools of *Aedes aegypti* species legs were tested. Of these 180 (25%) were caught in Chulaimbo, 123 (17%) in Kisumu, 178 (25%) in Msambweni, and 233 (33%) in Ukunda (Fig 2). The distribution of mosquito leg pools amongst each of the trap types was as follows: Ovitraps—248/714 pools (35.0%), Larval—193 (27.0%), Pupal—72 (10.1%), BGS—61 (8.5%), Prokopack—88 (12.3%) HLC—52 (7.3%) (Fig 2).

### Mosquito infection rates

**Fig 3:**
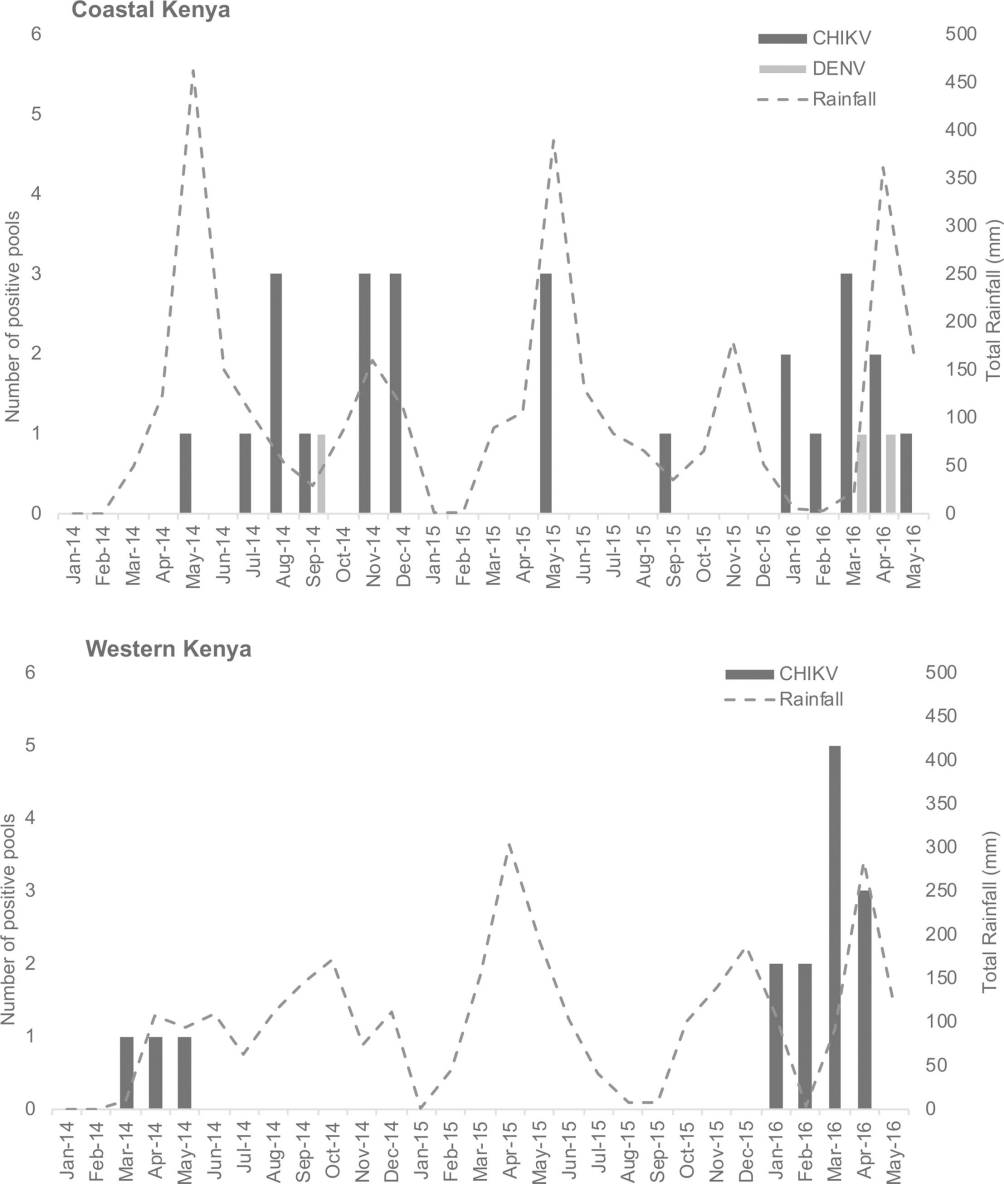
Temporal distribution of arbovirus positive mosquito leg pools and total rainfall across each study region. Mosquitoes and rainfall data collected between January 2014 and May 2016. Further study site details within western and coastal Kenya can be found in Fig 1.

Of the 714 pools tested, 43 (5.9%) were positive for CHIKV (Table 1, Fig 2) and 3 pools (0.4%) were positive for DENV, including 1 pool that was positive for both viruses (Table 2, Fig 2). ZIKV was not detected in in any mosquito pool tested in this study.

**Table 1:** Chikungunya. Poolscreen estimates for the minimum infection rate for CHIKV [16]. Kisumu and Ukunda represent the urban sites for the West and Coast, respectively. Rural collection sites were located in Chulaimbo and Msambweni for the West and Coast, respectively.

Sites	No. Pools	No. positive pools	Estimated infection rate (95% CI)
All	714	43	0.49% (0.34–0.68%)
West	321	17	0.33% (0.18–0.54%)
West rural	180	9	0.28% (0.12–0.53%)
West urban	123	8	0.42% (0.17–0.84%)
Coast	411	25	0.70% (0.43–1.10%)
Coast rural	178	13	0.85% (0.43–1.50%)
Coast urban	233	12	0.58% (0.28–1.00%)
All rural	369	22	0.46% (0.27–0.71%)
All urban	347	20	0.51% (0.29–0.81%)

**Table 2:** Dengue. Poolscreen estimates for the minimum infection rate for DENV [16]. Kisumu and Ukunda represent the urban sites for the West and Coast, respectively. Rural collection sites were located in Chulaimbo and Msambweni for the West and Coast, respectively.

Sites	No. pools	No. positive pools	Estimated infection rate (95% CI)
All	714	3	0.03% (0.01–0.10%)
West	321	0	0.00% (0.00–0.03%)
West rural	180	0	0.00% (0.00–0.06%)
West urban	123	0	0.00% (0.00–0.10%)
Coast	395	3	0.08% (0.02–0.24%)
Coast rural	178	2	0.13% (0.02–0.44%)
Coast urban	233	1	0.05% (0.00–0.25%)
All rural	369	2	0.04% (0.00–0.14%)
All urban	347	1	0.03% (0.00–0.13%)

### Spatiotemporal distribution

Of the 45 arbovirus-positive pools 17/45 (37.7%) were from mosquitoes from western Kenya and 28/45 (62.2%) were from the coast. All three of the DENV positive mosquito samples were from the coastal sites. The maximum-likelihood CHIKV infection rate for the coast was 0.7% (95% CI 0.43–1.10%), compared to 0.33% (95% CI 0.18% - 0.54%) in the west (Table 1). The infection rates for both CHIKV and DENV on the coast exceeded the upper confidence intervals for infection rate in the west, confirming regional variation in the circulation of CHIKV and DENV. Although the rural study sites of Chulaimbo and Msambweni yielded more arbovirus-positive pools than their urbanized counterparts, the calculated infection rates did not significantly differ between rural and urban sites (Tables 1 and 2).

CHIKV was identified in pools of both immature and mature mosquitoes and across all years and seasons of the study (Fig 2). CHIKV and DENV were not detected in western Kenya in the latter half of 2014 nor in 2015, although the detection of CHIKV resumed in the early part of 2016. On the coast, both viruses were more frequently detected throughout the study, with peaks of infected mosquitoes roughly corresponding to peak periods of rainfall (Fig 3). Of note, 22/42 (52.3%) of the CHIKV positive pools and 2/3 (66.6%) of the DENV positive samples were collected between January and May 2016.

### Life cycle stages

When grouped either as field-caught adult samples (those caught by BGS, HLC and Prokopack) or as immature form samples (caught by larval and pupal sampling or by ovitraps) the contribution of each group to the total number of positives is proportional to its size. Legs from adult mosquitoes accounted for 28% of the total sample pools tested (Table 3), and contributed 27.9% of all positive pools (Fig 2). Of the sample pools collected and tested, 72% were captured as immature mosquitoes and reared to adulthood prior to leg removal and pooling, contributing 72.1% of the positive pools. Due to the collection yield rates, adult pools were generally smaller (mean 7.2 individuals) than the overall study average pool size (15.2) and the immature form pool size (18.4). When expressed as a maximum likelihood infection rate [25], *i*.*e*., the estimated number of mosquitoes infected per 1,000 tested, the rate of CHIKV positivity was higher in adult mosquitoes (mean 61.8/1,000, range 30–100) than in immature mosquitoes (mean 9.0/1,000, range 2–24).

**Table 3:** The numbers of total *Ae*. *aegypti* pools caught by each collection method. The number of CHIKV positive pools of legs is shown in parentheses (). The number of DENV positives is shown in brackets [] The sex distribution of positive samples is given in the sub-rows. * One of the pools of mosquito legs from female mosquitoes was caught by HLC was positive for both viruses.

Collection type	No. of pools tested (Pos)	% of total pools	% of positive pools
BGS Sentinel	61 (4)	8.5	9.3
Male	1		
Female	3		
**Human Landing Catch**	52 **(3) [32]**	7.3	9.3
Male	0		
Female	3 [32] *		
**Prokopack Aspirator**	88 **(4)**	12.3	9.3
Male	1		
Female	3		
**Ovitrap**	248 **(10) [32]**	35.0	23.3
Male	5 [32]		
Female	5		
**Larval Collection**	193 **(9) [32]**	27.0	20.9
Male	4 [32]		
Female	5		
**Pupal Collection**	72 **(12)**	10.1	27.9
Male	6		
Female	5		
**Total**	**714 (43) [3]**	**100**	**100**

Ovitrap samples accounted for 35% of all pools tested, but with a proportionally smaller number of positives (23.3%). Conversely, pools of legs collected from pupal mosquitoes reared to adulthood contributed only 10.1% to the specimen total, but had 27.9% of positives. Indeed, the positivity rate in the larval collection was significantly increased in pairwise comparisons to both ovitraps (χ^2^ = 7.90, p = 0.005) and larval collection (χ^2^ = 10.35, p = 0.001). Rates for ovitraps and collected larvae did not significantly differ (χ^2^ = 0.12, p = 0.74). Likewise, there were no significant differences among the adult groups (χ^2^ = 0.63, p = 0.73) and no differences in positivity rates among adults and larvae (χ^2^ = 1.73, p = 0.79).

In our adult mosquito samples, 9/11 (81.8%) of positive samples were bloodmeal-seeking female mosquitoes, with one positive male pool each from the BGS sentinel traps and the Prokopack aspirator trapping. No male mosquitoes caught by HLC were positive for arboviruses. However, in the immature form samples, arbovirus positivity was relatively evenly distributed between sexes (Fig 2).

Sample pools from each sex and lifecycle stage were found to be positive for both DENV and CHIKV. Of note, two of the three DENV positive pools were from male adult mosquitoes that had been reared in the laboratory from eggs or larvae (one pool each) collected in Msambweni, suggesting that DENV was vertically transmitted to these mosquitoes. The third DENV positive pool were adult female mosquitoes caught by HLC in Ukunda, suggesting that DENV was continuing to circulate at a low level at that time.

## Discussion

Results of our study suggest that both DENV and CHIKV, but particularly CHIKV, remains in circulation at low levels in Kenyan vectors in both rural and urban settings. The viruses are maintained through all seasons, although peaks of infected mosquito abundance coincide with periods of increased rainfall. An association between rainfall and CHIKV incidence has been documented in other settings [26], but our detection of CHIKV in immature mosquitoes across all years and seasons of the study now indicates that this virus can be maintained by vertical transmission mechanisms during environmental conditions that are adverse for mosquito proliferation. While evidence of DENV TOT in Kenya has recently been described during epidemic times [7], the present study is the first to describe vertical transmission of CHIKV in wild *Ae*. *aegypti* mosquitoes and DENV during interepidemic times in Africa.

Extensive evidence for the maintenance of DENV and CHIKV by TOT mechanisms in endemic regions illustrates the stability of the DENV and CHIKV in wild mosquito populations (S1 Table). Evidence from experimental infections of *Aedes* spp. mosquitoes with CHIKV has demonstrated the potential for transovarial transmission of the virus [27–29]. Experimental evidence of TOT of CHIKV was debated in the 1980s [30], but has been repeatedly observed in laboratory settings with *Aedes* spp. (S1 Table) [27–29, 31–34]. The isolation of CHIKV viral RNA (but not viable virus) in artificially infected *Aedes aegypti* has been previously reported by Wong *et al*. [29]. In contrast Vazeille *et al*. reported on the absence of vertical transmission of the East/Central/South African (ECSA) strain of the virus in *Aedes albopictus* in their studies of experimental infections [35]. CHIKV TOT has been confirmed in wild mosquito populations recently, with a report by Jain *et al*. identifying CHIKV in field-caught immature *Aedes aegypti* mosquitoes in India [36], and a report by Thavara *et al*. finding CHIKV in *Aedes aegypti* and *Aedes albopictus* adult males in Thailand [37], both providing further evidence of vertical transmission in nature.

Our findings suggested the MIR for CHIKV was higher in adult mosquitoes as compared to immature mosquitoes. This might be expected if, in addition to vertical transmission of the virus (as suggested by our findings), adults may have also taken blood meals from CHIKV-positive humans. In addition, venereal infection of females by virus-positive male mosquitoes has been described as another potential route for infection of adult females [33]. Interestingly, the rate of infection of mosquitoes caught in larval collection was significantly higher than in the ovitraps. Bara et al. [38] suggest a mechanism of horizontal DENV transfer in *Aedes* mosquitoes, by which larvae may became infected by the ingestion of material from dead, vertically-infected immature mosquitoes or dead, infected adults [39, 40], yet such suggestions have yet to be documented with published data. In addition to its potential contribution to viral persistence, this mechanism may explain the proportionally increased rate of infection in our pupal samples.

We detected DENV in immature mosquito specimens and adult male pools, suggesting transovarial transmission of the virus in our samples. Thorough investigations have described DENV TOT in wild mosquitoes in a many affected geographic regions [7, 41–63] (S1 Table), suggesting an important role in the interepidemic maintenance of DENV. Although TOT mechanisms are thought not to be an important method of viral transmission of DENV in SE Asia and the Americas [15], the significant genetic divergence of *Ae*. *aegypti* mosquitoes from these regions compared to those in Africa has been documented [64]. Thus, it is possible that transovarial transmission mechanisms are of greater importance to DENV persistence in Africa compared to the rest of the tropics. Moreover, differences in the vertical transmission rate of DENV between *Aedes aegypti* and *Aedes albopictus* have been suggested previously [15], hence it is likely that that mosquito species also plays a defining role in the rate of vertical transmission of CHIKV. Future research comparing vertical transmission rates of different arboviruses in competent vector species could help to inform local vector and risk management policies. In future mosquito collections, we will endeavor to differentiate between different *Aedes* species in order to expand the understanding of species-specific TOT contributions to the global arboviral burden.

The increased presence of CHIKV in coastal Kenya, as observed in this study, is in agreement with previous studies that have shown high rates of alphavirus transmission in this region [65]. In addition, we only detected DENV-infected mosquitoes in the coastal study sites. This finding is in accord with previous epidemiologic findings on the regional variation in incidence of flavivirus infections in Kenya [12, 66–68]. During our 2014 to 2016 sampling period described in this study, we were also simultaneously collecting blood samples from child participants within acutely ill and healthy cohorts as a part of a larger, longitudinal study aiming to determine the incidence and prevalence of DENV and CHIKV in children in coastal and western Kenya, which has been previously described [10, 69]. Briefly, a primary diagnosis of acute DENV or CHIKV infection accounted for 24% (n = 93/385 participants) of the participants tested by RT-PCR [69]. These data specifically highlight the incidence of clinical CHIKV and DENV disease in Kenyan children, with a greater frequency of confirmed incident CHIKV cases from western Kenya than from the coast [69]. This is the opposite of the viral prevalence in mosquitoes that we have reported here. It is possible that CHIKV is persistently maintained in the mosquito population via transovarial transmission, particularly in regions where a larger proportion of the human population have some immunity to the virus. Moreover, in such regions of relatively higher immunity, local residents are hence less likely to present with symptomatic infections [70, 71]. We have previously noted that the presence of CHIKV in coastal Kenya may be misinterpreted by the presence of a closely related alphavirus, O’nyong’nyong virus [65], which is transmitted by anopheline mosquitoes [65], yet indistinguishable from CHIKV when utilizing common serological diagnostic tools due to antibody cross-reactivity [72]. Yet, repeated discovery of arboviruses in competent vectors and in human populations reiterates the public health importance of these findings, and the need for better integration into local vector control measures and community education.

Our study had limitations. An inherent sampling bias existed in our study due to the relative difficulty in capturing adult mosquitoes compared to eggs and larvae. We have, however adjusted for this by expressing our data proportionally and, where relevant, as minimum infective rate. As with all studies, it is possible that some variation was introduced by the different trap operators and entomologists at the different study sites. We minimized such effects by ensuring that all personnel were briefed and trained together, by rotating staff between the 4 sites, and by holding regular conference calls between all staff to ensure consistency of our protocols and methodologies. This study focused on the detection of viral RNA from pooled mosquito legs, and data presented in this manuscript is not representative of potential live virus that may have been contained within our samples. We were unable to recover viable CHIKV and DENV in the laboratory as the test samples were stored in a lysis buffer, which rendered them unsuitable for inoculation into cell culture. However, we did confirm the presence of CHIKV in a subset of our samples using individual monoplex reactions, using the same primers as used in the multiplex reaction. The template used in these confirmatory monoplex assays was generated in a separate reaction to that used for the multiplex assay. Additionally, initial mosquito specimen processing occurred in laboratories that did not contain any live virus, and molecular testing was conducted in a laboratory in which structural measures including a one-way work flow and separate room for nucleic acid isolation and detection are instituted to mitigate the risk of contamination. Thus, we are confident that our positive findings are not the result of cross-contamination. Further confirmation of vertical transmission of CHIKV is important to the elucidation of the mechanisms by which the virus is sustained outside of its human host. To achieve this, future research should involve the appropriate collection and storage of mosquito samples so that live virus, when present, can be isolated and characterized.

Our findings highlight the health implications of regional variation in arboviral circulation, and underscore the importance of understanding mechanisms of arboviral transmission to local risk assessment and healthcare management. Importantly, we have provided evidence that CHIKV and DENV are vertically transmitted in *Ae*. *aegypti* mosquitoes, likely facilitating the maintenance of baseline levels of virus in the study locations. Low-level circulation may explain the lack of seasonal patterns in CHIKV and DENV epidemics in Kenya, yet regularly occurring TOT is likely to contribute to local viral maintenance and a continuous, low-level incidence of human infections that establish a higher baseline immunity to these viruses within the local population.

## Supporting information

S1 Table A summary of published evidence of TOT reported in both wild and experimental environments. NS = Not Specified.
(XLSX)

## References

[1] Okello GBA AN, Ouma J, Cherogony SC, Tukei PM, Ochieng W, Den Boer JW, Sanders EJ. Outbreak of yellow fever in Kenya. The Lancet. 1993;341(8843):489. 10.1016/0140-6736(93)90237-b 8094503

[2] Sanders EJ, Marfin AA, Tukei PM, Kuria G, Ademba G, Agata NN, et al First recorded outbreak of yellow fever in Kenya, 1992–1993. I. Epidemiologic investigations. Am J Trop Med Hyg. 1998;59(4):644–9. 10.4269/ajtmh.1998.59.644 9790446

[3] Sergon K, Njuguna C, Kalani R, Ofula V, Onyango C, Konongoi LS, et al Seroprevalence of Chikungunya virus (CHIKV) infection on Lamu Island, Kenya, October 2004. Am J Trop Med Hyg. 2008;78(2):333–7. 18256441

[4] Mwaengo D, Lorenzo G, Iglesias J, Warigia M, Sang R, Bishop RP, et al Detection and identification of Rift Valley fever virus in mosquito vectors by quantitative real-time PCR. Virus Res. 2012;169(1):137–43. 10.1016/j.virusres.2012.07.019 22841800

[5] Woods CW, Karpati AM, Grein T, McCarthy N, Gaturuku P, Muchiri E, et al An outbreak of Rift Valley fever in Northeastern Kenya, 1997–98. Emerg Infect Dis. 2002;8(2):138–44. 10.3201/eid0802.010023 11897064 PMC2732454

[6] Center for Disease Control and Prevention. Rift Valley fever outbreak—Kenya, November 2006—January 2007. Morb Mortal Wkly Rep. 2007;56(4):73–6. 17268404

[7] Lutomiah J, Barrera R, Makio A, Mutisya J, Koka H, Owaka S, et al Dengue Outbreak in Mombasa City, Kenya, 2013–2014: Entomologic Investigations. PLoS Negl Trop Dis. 2016;10(10):e0004981 10.1371/journal.pntd.0004981 27783626 PMC5082659

[8] Grossi-Soyster EN, Cook EAJ, de Glanville WA, Thomas LF, Krystosik AR, Lee J, et al Serological and spatial analysis of alphavirus and flavivirus prevalence and risk factors in a rural community in western Kenya. PLoS Negl Trop Dis. 2017;11(10):e0005998 10.1371/journal.pntd.0005998 29040262 PMC5659799

[9] Gudo ES, Ali S, Antonio VS, Chelene IR, Chongo I, Demanou M, et al Seroepidemiological Studies of Arboviruses in Africa. Adv Exp Med Biol. 2018;1062:361–71. 10.1007/978-981-10-8727-1_25 29845545

[10] Hortion J, Mutuku FM, Eyherabide AL, Vu DM, Boothroyd DB, Grossi-Soyster EN, et al Acute Flavivirus and Alphavirus Infections among Children in Two Different Areas of Kenya, 2015. Am J Trop Med Hyg. 2019;100(1):170–3. 10.4269/ajtmh.18-0297 30457092 PMC6335892

[11] LaBeaud AD, Sutherland LJ, Muiruri S, Muchiri EM, Gray LR, Zimmerman PA, et al Arbovirus prevalence in mosquitoes, Kenya. Emerg Infect Dis. 2011;17(2):233–41. 10.3201/eid1702.091666 21291594 PMC3204744

[12] Vu DM, Banda T, Teng CY, Heimbaugh C, Muchiri EM, Mungai PL, et al Dengue and West Nile Virus Transmission in Children and Adults in Coastal Kenya. Am J Trop Med Hyg. 2017;96(1):141–3. 10.4269/ajtmh.16-0562 27821697 PMC5239681

[13] Vu DM, Mutai N, Heath CJ, Vulule JM, Mutuku FM, Ndenga BA, et al Unrecognized Dengue Virus Infections in Children, Western Kenya, 2014–2015. Emerg Infect Dis. 2017;23(11):1915–7. 10.3201/eid2311.170807 29048283 PMC5652413

[14] Diniz DFA, de Albuquerque CMR, Oliva LO, de Melo-Santos MAV, Ayres CFJ. Diapause and quiescence: dormancy mechanisms that contribute to the geographical expansion of mosquitoes and their evolutionary success. Parasit Vectors. 2017;10(1):310 10.1186/s13071-017-2235-0 28651558 PMC5485599

[15] Grunnill M, Boots M. How important is vertical transmission of dengue viruses by mosquitoes (*Diptera*: *Culicidae*)? J Med Entomol. 2016;53(1):1–19. 10.1093/jme/tjv168 26545718

[16] Ebert D, Herre EA. The evolution of parasitic diseases. Parasitol Today. 1996;12(3):96–101. 10.1016/0169-4758(96)80668-5 15275238

[17] Lambrechts L, Scott TW. Mode of transmission and the evolution of arbovirus virulence in mosquito vectors. Proc Biol Sci. 2009;276(1660):1369–78. 10.1098/rspb.2008.1709 19141420 PMC2660968

[18] Ndenga BA, Mutuku FM, Ngugi HN, Mbakaya JO, Aswani P, Musunzaji PS, et al Characteristics of Aedes aegypti adult mosquitoes in rural and urban areas of western and coastal Kenya. PLoS One. 2017;12(12):e0189971 10.1371/journal.pone.0189971 29261766 PMC5736227

[19] Ngugi HN, Mutuku FM, Ndenga BA, Musunzaji PS, Mbakaya JO, Aswani P, et al Characterization and productivity profiles of Aedes aegypti (L.) breeding habitats across rural and urban landscapes in western and coastal Kenya. Parasit Vectors. 2017;10(1):331 10.1186/s13071-017-2271-9 28701194 PMC5508769

[20] Vazquez-Prokopec GM, Galvin WA, Kelly R, Kitron U. A new, cost-effective, battery-powered aspirator for adult mosquito collections. J Med Entomol. 2009;46(6):1256–9. 10.1603/033.046.0602 19960668 PMC2800949

[21] Chadee DD, Doon R, Severson DW. Surveillance of dengue fever cases using a novel Aedes aegypti population sampling method in Trinidad, West Indies: the cardinal points approach. Acta Trop. 2007;104(1):1–7. 10.1016/j.actatropica.2007.06.006 17803949

[22] Gillett J, Smith J. Common African mosquitoes and their medical importance. London: William Heinemann Medical Books Limited; 1972.

[23] Waggoner JJ, Gresh L, Mohamed-Hadley A, Ballesteros G, Davila MJ, Tellez Y, et al Single-Reaction Multiplex Reverse Transcription PCR for Detection of Zika, Chikungunya, and Dengue Viruses. Emerg Infect Dis. 2016;22(7):1295–7. 10.3201/eid2207.160326 27184629 PMC4918162

[24] Waggoner JJ, Ballesteros G, Gresh L, Mohamed-Hadley A, Tellez Y, Sahoo MK, et al Clinical evaluation of a single-reaction real-time RT-PCR for pan-dengue and chikungunya virus detection. J Clin Virol. 2016;78:57–61. 10.1016/j.jcv.2016.01.007 26991052 PMC4836994

[25] Katholi CR, Unnasch TR. Important experimental parameters for determining infection rates in arthropod vectors using pool screening approaches. Am J Trop Med Hyg. 2006;74(5):779–85. 16687680

[26] Wiwanitkit S aWV. Chikungunya virus infection and relationship to rainfall, the relationship study from southern Thailand. Journal of Arthropod-Borne Diseases. 2013;7(2):185–7. 24409444 PMC3875885

[27] Agarwal A, Dash PK, Singh AK, Sharma S, Gopalan N, Rao PV, et al Evidence of experimental vertical transmission of emerging novel ECSA genotype of Chikungunya Virus in Aedes aegypti. PLoS Negl Trop Dis. 2014;8(7):e2990 10.1371/journal.pntd.0002990 25080107 PMC4117456

[28] Chompoosri J, Thavara U, Tawatsin A, Boonserm R, Phumee A, Sangkitporn S, et al Vertical transmission of Indian Ocean Lineage of chikungunya virus in Aedes aegypti and Aedes albopictus mosquitoes. Parasit Vectors. 2016;9:227 10.1186/s13071-016-1505-6 27108077 PMC4842298

[29] Wong HV, Vythilingam I, Sulaiman WY, Lulla A, Merits A, Chan YF, et al Detection of Persistent Chikungunya Virus RNA but not Infectious Virus in Experimental Vertical Transmission in *Aedes aegypti* from Malaysia. Am J Trop Med Hyg. 2016;94(1):182–6. 10.4269/ajtmh.15-0318 26598564 PMC4710427

[30] Mourya DT. Absence of transovarial transmission of Chikungunya virus in *Aedes aegypti* & *Ae*. *albopictus* mosquitoes. Indian J Med Res. 1987;85:593–5. 3666861

[31] Hailin Z YZ, Zhuqing M. Transovarial transmission of chikungunya virus in *Aedes albopictus* and *Aedes aegypti* mosquitoes. Chin J Virol. 1993;9:222–7.

[32] Honorio NA, Wiggins K, Eastmond B, Camara DCP, Alto BW. Experimental Vertical Transmission of Chikungunya Virus by Brazilian and Florida Aedes Albopictus Populations. Viruses. 2019;11(4). 10.3390/v11040353 PMC6520672 30999594

[33] Mavale M, Parashar D, Sudeep A, Gokhale M, Ghodke Y, Geevarghese G, et al Venereal transmission of chikungunya virus by *Aedes aegypti* mosquitoes (Diptera: Culicidae). Am J Trop Med Hyg. 2010;83(6):1242–4. 10.4269/ajtmh.2010.09-0577 21118928 PMC2990038

[34] Zytoon EM, el-Belbasi HI, Matsumura T. Transovarial transmission of chikungunya virus by *Aedes albopictus* mosquitoes ingesting microfilariae of *Dirofilaria immitis* under laboratory conditions. Microbiol Immunol. 1993;37(5):419–21. 10.1111/j.1348-0421.1993.tb03232.x 8394983

[35] Vazeille M, Mousson L, Failloux AB. Failure to demonstrate experimental vertical transmission of the epidemic strain of Chikungunya virus in *Aedes albopictus* from La Reunion Island, Indian Ocean. Mem Inst Oswaldo Cruz. 2009;104(4):632–5. 10.1590/s0074-02762009000400017 19722089

[36] Jain J, Kushwah RBS, Singh SS, Sharma A, Adak T, Singh OP, et al Evidence for natural vertical transmission of chikungunya viruses in field populations of *Aedes aegypti* in Delhi and Haryana states in India-a preliminary report. Acta Trop. 2016;162:46–55. 10.1016/j.actatropica.2016.06.004 27282096

[37] Thavara U, Tawatsin A, Pengsakul T, Bhakdeenuan P, Chanama S, Anantapreecha S, et al Outbreak of chikungunya fever in Thailand and virus detection in field population of vector mosquitoes, *Aedes aegypti* (L.) and *Aedes albopictus Skuse* (*Diptera*: *Culicidae*). Southeast Asian J Trop Med Public Health. 2009;40(5):951–62. 19842379

[38] Bara JJ, Clark TM, Remold SK. Susceptibility of larval Aedes aegypti and Aedes albopictus (Diptera: Culicidae) to dengue virus. J Med Entomol. 2013;50(1):179–84. 10.1603/me12140 23427668

[39] Miller BR, DeFoliart GR, Hansen WR, Yuill TM. Infection rates of Aedes triseriatus following ingestion of La Crosse virus by the larvae. Am J Trop Med Hyg. 1978;27(3):605–8. 10.4269/ajtmh.1978.27.605 27990

[40] Turell MJ, Linthicum KJ, Beaman JR. Transmission of Rift Valley fever virus by adult mosquitoes after ingestion of virus as larvae. Am J Trop Med Hyg. 1990;43(6):677–80. 10.4269/ajtmh.1990.43.677 2267972

[41] Angel A, Angel B, Joshi V. Rare occurrence of natural transovarial transmission of dengue virus and elimination of infected foci as a possible intervention method. Acta Trop. 2016;155:20–4. 10.1016/j.actatropica.2015.11.018 26655042

[42] Angel B, Joshi V. Distribution and seasonality of vertically transmitted dengue viruses in Aedes mosquitoes in arid and semi-arid areas of Rajasthan, India. J Vector Borne Dis. 2008;45(1):56–9. 18399318

[43] Arunachalam N TS, Thenmozhi V, Rajendran R, Paramasivan R, Manavalan R, Ayanar K, Tyagi BK. Natural vertical transmission of dengue viruses by *Aedes aegypti* in Chennai, Tamil Nadu, India. Indian J Med Res. 2008;127(4):395–7. 18577796

[44] Cecilio SG, Junior WF, Totola AH, de Brito Magalhaes CL, Ferreira JM, de Magalhaes JC. Dengue virus detection in Aedes aegypti larvae from southeastern Brazil. J Vector Ecol. 2015;40(1):71–4. 10.1111/jvec.12134 26047186

[45] Cruz LC, Serra OP, Leal-Santos FA, Ribeiro AL, Slhessarenko RD, Santos MA. Natural transovarial transmission of dengue virus 4 in Aedes aegypti from Cuiaba, State of Mato Grosso, Brazil. Rev Soc Bras Med Trop. 2015;48(1):18–25. 10.1590/0037-8682-0264-2014 25860459

[46] da Costa CF, Dos Passos RA, Lima JBP, Roque RA, de Souza Sampaio V, Campolina TB, et al Transovarial transmission of DENV in Aedes aegypti in the Amazon basin: a local model of xenomonitoring. Parasit Vectors. 2017;10(1):249 10.1186/s13071-017-2194-5 28526066 PMC5437422

[47] de Figueiredo ML, de CGA, Amarilla AA, de SLA, de SOA, de Araujo RF, et al Mosquitoes infected with dengue viruses in Brazil. Virol J. 2010;7:152 10.1186/1743-422X-7-152 20624314 PMC2913956

[48] Dutta P, Khan SA, Chetry S, Dev V, Sarmah CK, Mahanta J. First evidence of dengue virus infection in wild caught mosquitoes during an outbreak in Assam, Northeast India. J Vector Borne Dis. 2015;52(4):293–8. 26714508

[49] Guedes DR, Cordeiro MT, Melo-Santos MA, Magalhaes T, Marques E, Regis L, et al Patient-based dengue virus surveillance in *Aedes aegypti* from Recife, Brazil. J Vector Borne Dis. 2010;47(2):67–75. 20539043

[50] Günther J, Martinez-Munoz JP, Perez-Ishiwara DG, Salas-Benito J. Evidence of vertical transmission of dengue virus in two endemic localities in the state of Oaxaca, Mexico. Intervirology. 2007;50(5):347–52. 10.1159/000107272 17700030

[51] Gutierrez-Bugallo G, Rodriguez-Roche R, Diaz G, Vazquez AA, Alvarez M, Rodriguez M, et al First record of natural vertical transmission of dengue virus in Aedes aegypti from Cuba. Acta Trop. 2017;174:146–8. 10.1016/j.actatropica.2017.07.012 28720490

[52] Hull B, Tikasingh E, de Souza M, Martinez R. Natural transovarial transmission of dengue 4 virus in *Aedes aegypti* in Trinidad. Am J Trop Med Hyg. 1984;33(6):1248–50. 10.4269/ajtmh.1984.33.1248 6542323

[53] Khin MM, Than KA. Transovarial transmission of dengue 2 virus by *Aedes aegypti* in nature. Am J Trop Med Hyg. 1983;32(3):590–4. 10.4269/ajtmh.1983.32.590 6859404

[54] Martinez NE, Dzul-Manzanilla F, Gutierrez-Castro C, Ibarra-Lopez J, Bibiano-Marin W, Lopez-Damian L, et al Natural vertical transmission of dengue-1 virus in *Aedes aegypti* populations in Acapulco, Mexico. J Am Mosq Control Assoc. 2014;30(2):143–6. 10.2987/14-6402.1 25102601

[55] Martins VE, Alencar CH, Kamimura MT, de Carvalho Araujo FM, De Simone SG, Dutra RF, et al Occurrence of natural vertical transmission of dengue-2 and dengue-3 viruses in *Aedes aegypti* and *Aedes albopictus* in Fortaleza, Ceara, Brazil. PLoS One. 2012;7(7):e41386 10.1371/journal.pone.0041386 22848479 PMC3405123

[56] Rohani A, Aidil Azahary AR, Malinda M, Zurainee MN, Rozilawati H, Wan Najdah WM, et al Eco-virological survey of Aedes mosquito larvae in selected dengue outbreak areas in Malaysia. J Vector Borne Dis. 2014;51(4):327–32. 25540966

[57] Serufo JC, de Oca HM, Tavares VA, Souza AM, Rosa RV, Jamal MC, et al Isolation of dengue virus type 1 from larvae of *Aedes albopictus* in Campos Altos city, State of Minas Gerais, Brazil. Mem Inst Oswaldo Cruz. 1993;88(3):503–4. 10.1590/s0074-02761993000300025 8107613

[58] Thavara U, Siriyasatien P, Tawatsin A, Asavadachanukorn P, Anantapreecha S, Wongwanich R, et al Double infection of heteroserotypes of dengue viruses in field populations of *Aedes aegypti* and *Aedes albopictus* (Diptera: Culicidae) and serological features of dengue viruses found in patients in southern Thailand. Southeast Asian J Trop Med Public Health. 2006;37(3):468–76. 17120966

[59] Thenmozhi V, Tewari SC, Manavalan R, Balasubramanian A, Gajanana A. Natural vertical transmission of dengue viruses in *Aedes aegypt* in southern India. Trans R Soc Trop Med Hyg. 2000;94(5):507 10.1016/s0035-9203(00)90067-1 11132376

[60] Thongrungkiat S, Maneekan P, Wasinpiyamongkol L, Prummongkol S. Prospective field study of transovarial dengue-virus transmission by two different forms of *Aedes aegypti* in an urban area of Bangkok, Thailand. J Vector Ecol. 2011;36(1):147–52. 10.1111/j.1948-7134.2011.00151.x 21635652

[61] Thongrungkiat S, Wasinpiyamongkol L, Maneekan P, Prummongkol S, Samung Y. Natural transovarial dengue virus infection rate in both sexes of dark and pale forms of *Aedes aegypti* from an urban area of Bangkok, Thailand. Southeast Asian J Trop Med Public Health. 2012;43(5):1146–52. 23431820

[62] Velandia-Romero ML, Olano VA, Coronel-Ruiz C, Cabezas L, Calderon-Pelaez MA, Castellanos JE, et al Dengue virus detection in *Aedes aegypti* larvae and pupae collected in rural areas of Anapoima, Cundinamarca, Colombia. Biomedica. 2017;37(0):193–200. 10.7705/biomedica.v37i0.3584 29161491

[63] Zeidler JD, Acosta PO, Barreto PP, Cordeiro Jda S. Dengue virus in *Aedes aegypti* larvae and infestation dynamics in Roraima, Brazil. Rev Saude Publica. 2008;42(6):986–91. 10.1590/s0034-89102008005000055 19031506

[64] Brown JE, McBride CS, Johnson P, Ritchie S, Paupy C, Bossin H, et al Worldwide patterns of genetic differentiation imply multiple 'domestications' of Aedes aegypti, a major vector of human diseases. Proc Biol Sci. 2011;278(1717):2446–54. 10.1098/rspb.2010.2469 21227970 PMC3125627

[65] LaBeaud AD, Banda T, Brichard J, Muchiri EM, Mungai PL, Mutuku FM, et al High rates of o'nyong nyong and Chikungunya virus transmission in coastal Kenya. PLoS Negl Trop Dis. 2015;9(2):e0003436 10.1371/journal.pntd.0003436 25658762 PMC4319898

[66] Konongoi L, Ofula V, Nyunja A, Owaka S, Koka H, Makio A, et al Detection of dengue virus serotypes 1, 2 and 3 in selected regions of Kenya: 2011–2014. Virol J. 2016;13(1):182 10.1186/s12985-016-0641-0 27814732 PMC5097412

[67] Mease LE, Coldren RL, Musila LA, Prosser T, Ogolla F, Ofula VO, et al Seroprevalence and distribution of arboviral infections among rural Kenyan adults: a cross-sectional study. Virol J. 2011;8:371 10.1186/1743-422X-8-371 21794131 PMC3161961

[68] Sutherland LJ, Cash AA, Huang YJ, Sang RC, Malhotra I, Moormann AM, et al Serologic evidence of arboviral infections among humans in Kenya. Am J Trop Med Hyg. 2011;85(1):158–61. 10.4269/ajtmh.2011.10-0203 21734142 PMC3122361

[69] Waggoner J, Brichard J, Mutuku F, Ndenga B, Heath CJ, Mohamed-Hadley A, et al Malaria and chikungunya detected using molecular diagnostics among febrile Kenyan children. Open Form Infect Dis. 2017;4(3):oxf110. 10.1093/ofid/ofx110 PMC5505337 28702473

[70] Queyriaux B, Simon F, Grandadam M, Michel R, Tolou H, Boutin JP. Clinical burden of chikungunya virus infection. Lancet Infect Dis. 2008;8(1):2–3. 10.1016/S1473-3099(07)70294-3 18156079

[71] Sissoko D, Moendandze A, Malvy D, Giry C, Ezzedine K, Solet JL, et al Seroprevalence and risk factors of chikungunya virus infection in Mayotte, Indian Ocean, 2005–2006: a population-based survey. PLoS One. 2008;3(8):e3066 10.1371/journal.pone.0003066 18725980 PMC2518850

[72] Pezzi L, Reusken CB, Weaver SC, Drexler JF, Busch M, LaBeaud AD, et al GloPID-R report on Chikungunya, O'nyong-nyong and Mayaro virus, part I: Biological diagnostics. Antiviral Res. 2019;166:66–81. 10.1016/j.antiviral.2019.03.009 30905821

